# Analyzing historical trends in breast cancer biomarker expression: a feasibility study (1947–2009)

**DOI:** 10.1038/npjbcancer.2015.16

**Published:** 2015-10-07

**Authors:** Nancy Krieger, Laurel A Habel, Pamela D Waterman, Melina Shabani, Lis Ellison-Loschmann, Ninah S Achacoso, Luana Acton, Stuart J Schnitt

**Affiliations:** 1 Department of Social and Behavioral Sciences, Harvard T.H. Chan School of Public Health, Boston, MA, USA; 2 Kaiser Permanente Division of Research, Oakland, CA, USA; 3 Department of Pathology, Beth Israel Deaconess Medical Center, Harvard Medical School, Boston, MA, USA; 4 Centre for Public Health Research, Massey University, Wellington, New Zealand

## Abstract

**Background/Objectives:**

Determining long-term trends in tumor biomarker expression is essential for understanding aspects of tumor biology amenable to change. Limiting the availability of such data, currently used assays for biomarkers are relatively new. For example, assays for the estrogen receptor (ER), which are the oldest, extend back only to the 1970s.

**Methods:**

To extend scant knowledge about the feasibility of obtaining long-term data on tumor biomarkers, we randomly selected 60 breast cancer cases (10 per decade) diagnosed between 1947–2009 among women members of the Kaiser Permanente Northern California health plan to obtain and analyze their formalin-fixed paraffin-embedded (FFPE) tumor specimens. For each tumor specimen, we created duplicate tissue microarrays for analysis.

**Results:**

We located tumor blocks and pathology reports for 50 of the 60 cases (83%), from which we randomly sampled 5 cases per decade for biomarker analysis (*n*=30). All 30 cases displayed excellent morphology and exhibited biomarkers compatible with histologic type and grade. Test–retest reliability was also excellent: 100% for ER; 97% for human epidermal growth factor receptor 2 and epidermal growth factor receptor; 93% for progesterone receptor and cytokeratin 5/6; and 90% for Ki67 and molecular phenotype; the kappa statistic was excellent (>0.9) for 4 of the 7 biomarkers, strong (0.6–0.8) for 2, and fair for only 1 (owing to low prevalence).

**Conclusions:**

These results indicate immunostaining for biomarkers commonly used to evaluate breast cancer biology and assign surrogate molecular phenotypes can reliably be employed on archival FFPE specimens up to 60 years old.

## Introduction

Determining long-term trends in tumor biomarkers is crucial for understanding what aspects of tumor biology are amenable to change. Evidence of long-term trends critically complements cross-sectional comparisons, across geographical regions or social groups, because only long-term data can detect the impact of changing exogenous exposures.^1^ For example, the recent rise and fall in breast cancer incidence in many countries, linked to the rise and fall of postmenopausal hormone therapy use^2–9^ was paralleled by a rise and fall in the incidence of estrogen receptor positive (ER^+^) tumors.^3,4^ Of note, although breast cancers tumors are generally more often ER^+^ among US white compared with black women,^10^ the US white/black odds ratio for ER^+^ breast tumors nevertheless exhibited a parallel rise and fall during this same time period, a pattern likely attributable to changes in hormone therapy use.^11^

Scant knowledge, however, exists about the feasibility of locating and analyzing decades old, population-based archival formalin-fixed paraffin-embedded (FFPE) tumor specimens. Contributing to the lack of such historical data is the relatively recent development of most currently used assays: in the case of breast cancer, for example, one of the first such assays, for ER, became available only in the 1970s,^12^ and characterization of molecular phenotypes is an innovation of the 21st century CE.^13^ We accordingly conducted a novel feasibility study, including assessment of test–retest reliability, for a series of breast cancer cases spanning 6 decades (1947–2009). Favorable results would enhance interpretation of prior studies that have employed biomarker immunostains on old FFPE specimens, e.g., 30–40 years old when analyzed,^14,15^ as well as encourage new research.

## Materials and Methods

For our study, we analyzed FFPE specimens obtained from women diagnosed with invasive breast cancer who were members of Kaiser Permanente, Northern California (KPNC; institutional review board approval: Harvard School of Public Health/#CR-20929–02; KPNC/#CN-13LHabe-03-H). KPNC is an integrated healthcare delivery system established in the 1940s^16^ and whose cancer registry dates back to 1947.^17^ Since its inception as a health plan for workers employed in World War II shipyards, KPNC’s membership has ranged from working class to professional and has mirrored the well-known diversity of the San Francisco Bay Area and Central Valley, comprised of white, black, Hispanic, Asian and Pacific Islander, and American Indian populations.^17,18^

Among the 60,904 breast cancer cases diagnosed between 1947 and 2009, 7,150 met our feasibility study’s eligibility criteria: 50–64 years old at diagnosis and invasive tumor⩾1 cm. We randomly selected 10 eligible cases per each of the 6 time periods (hereafter referred to as decades: 1947–1959; 1960–1969;…; 2000–2009), and used information available as of 1987 to restrict sampling to cases with lymph node positive tumors. Our rationale was to maximize the chance that biomarkers would be positive or credibly negative, given that advanced cases are more likely to be positive for human epidermal growth factor receptor 2 (HER2), epidermal growth factor receptor (EGFR), high Ki67, ER^−^, and PR^−^.^13,19^ Thus, had the specimens included only early stage cancer, it would be less clear if negative test results could be interpreted as truly negative—versus falsely negative—because the assay was not sensitive to biomarker expression in the older specimens. Among the 50 cases located with eligible blocks containing tumor (as described below), and in accord with our *a priori* power calculations, we selected a random sample of 5 cases per decade (total *n*=30) for biomarker immunohistochemical analysis.

To conduct the assays, we first created tissue microarrays (TMAs) in duplicate, each employing 3, 0.6-mm cores per specimen. We assessed antigen preservation by using a cytokeratin (CK) “cocktail” immunostain (consisting of antibodies AE1/AE3 and Cam5.2, which together recognize a broad spectrum of CKs), and also performed immunostains for biomarkers routinely used to assess breast cancer biology and assign molecular phenotype based on surrogate markers^13,19–22^: ER, progesterone receptor (PR), HER2, CK 5/6, EGFR, and Ki67. The Ki67 stains were quantitated by computer-assisted image analysis;^23^ for each case, the score equaled the average percentage of positive cells per core. Review of the biomarkers was blinded to tumor histologic type and grade, and review of each set of TMA results was independent and blinded to the other. For each TMA, if a tumor marker was scored as positive for 1 or more cores, it received an overall rating of positive for that marker.

## Results

Among the random sample of 60 selected cases, we located pathology reports for 55 cases (92%), of which 50 (83% of the 60) had blocks that contained tumor tissue. Among the random sample of 5 cases per decade selected from these 50 cases, notably all 30 cases (100%) displayed excellent morphology and the CK cocktail staining results (Table 1) indicated antigen integrity was preserved for all but 2 of the cases (1 from the 1950s, 1 from the 1960s). Test–retest reliability was likewise excellent (Table 1): 100% for ER, 97% for HER2 and EGFR, 93% for PR and CK 5/6, and 90% for Ki67 (scored as <14% vs. ⩾14%^19^). The kappa statistic (taking into account chance agreement;^24^ Table 1) was excellent (>0.9) for 4 of the 7 biomarkers, moderate-to-strong (0.6–0.8) for 2, and fair for 1 (0.346 for Ki67 as a dichotomous variable). On the basis of the biomarker results, concordance for molecular phenotype was 90% (kappa >0.7): classification of 15 cases was concordant for Luminal A, 3 for Luminal B, 3 for basal-like, 3 for HER2, and 2 for “unclassified”; only 3 cases were discordant for classification as either Luminal A or B.

Assay results indicated that ~80% of tumors were ER^+^ (i.e., 80% for all decades except for the 1960s, for which 3/5 (60%) of the cases were ER^+^), all ER^−^ tumors were grade 3, and virtually all ER^+^ tumors were grade 1 or grade 2. For PR, 60% of tumors were PR^+^ and all but one of the PR^−^ tumors was grade 3 (the exception was grade 1). In addition, most tumors were negative for: HER2 (60–80% negative); CK 5/6 (87% negative); and EGFR (93% negative); Ki67 was <14% for 86–90% of the cases.

## Discussion

Considered together, our findings provide promising evidence for investigators seeking to conduct analyses of historical trends in tumor characteristics. First, perhaps unique to KPNC, we were able to locate pathology reports and informative tumor blocks for 83% of cases diagnosed between 1947 and 2009, with no discernible difference in specimen retrieval by decade. Second, more generalizably, we demonstrated that, among a random sample of breast cancer tumor specimens spanning this time period, current immunostaining methods for biomarkers commonly used to evaluate breast cancer biology and assign molecular subtype^13,19–22^ yielded plausible results for both old and recent specimens,^13,19^ with excellent test–retest reliability. Of note, the lower test–retest reliability (93%) observed for both PR and CK 5/6 is consistent with prior studies indicating that expression of these biomarkers may demonstrate intratumor heterogeneity,^21,22^ regardless of the assay used. The high concordance (90%) but low kappa (0.346) for Ki67 reflects the low prevalence of high values.^24^

In summary, our study provides novel evidence that current immunostain techniques can feasibly and reliably be used on old FFPE specimens, dating back 60 years. It accordingly suggests that prior^14,15^ and future studies employing immunostains to characterize long-term trends in tumor biomarker expression at the population level can yield credible results.

## Figures and Tables

**Table 1:** Immunostain assay results, based on two independent TMAs 1 and 2, for 30 randomly selected breast cancer tumor specimens (5 per decade; all FFPE) for: CK cocktail, ER, PR, HER2, CK 5/6, EGFR, Ki67, and molecular phenotype (Kaiser Permanente, Northern California, USA, 1947–2009)

*Year range*	*ID*	*Year of diagnosis*	*Histologic diagnosis*	*Histologic grade*	*CK cocktail*		*ER*		*PR*		*HER2*		*CK 5/6*		*EGFR*		*Ki67*		*Molecular phenotype*	
					*TMA1*	*TMA2*	*TMA1*	*TMA2*	*TMA1*	*TMA2*	*TMA1*	*TMA2*	*TMA1*	*TMA2*	*TMA1*	*TMA2*	*TMA1*	*TMA2*	*TMA1*	*TMA2*
1947–1959	1–1	1955	Invasive carcinoma with ductal and lobular features	2	+	+	+	+	−	−	−	−	−	−	−	−	1.3%	0.3%	Luminal A	Luminal A
	1–2	1957	IDC	2	+	+	+	+	+	+	3+	3+	−	−	−	−	9.0%	4.7%	Luminal B	Luminal B
	1–3	1959	IDC	3	+	+	+	+	+	+	−	−	−	−	−	−	2.7%	5.3%	Luminal A	Luminal A
	1–4	1956	Invasive lobular carcinoma	2	+	+	+	+	+	+	−	−	−	−	−	−	1.3%	2.0%	Luminal A	Luminal A
	1–5	1956	IDC	3	−	−	−	−	−	−	−	−	−	−	−	−	12.3%	12.7%	Unclassifiable	Unclassifiable
1960–1969	2–6	1968	Solid papillary carcinoma	2	+	+	+	+	+	+	−	−	−	−	−	−	3.0%	3.3%	Luminal A	Luminal A
	2–7	1969	IDC	3	+	+	−	−	−	−	2+	2+	−	−	+	−	23.0%	18.0%	Basal-like	Unclassifiable
	2–8	1969	IDC	2	+	+	+	+	+	+	3+	3+	−	−	−	−	9.0%	5.7%	Luminal B	Luminal B
	2–9	1968	Invasive mucinous carcinoma	2	−	−	+	+	+	+	−	−	−	−	−	−	2.7%	4.0%	Luminal A	Luminal A
	2–10	1965	IDC with medullary features	3	+	+	−	−	−	−	−	−	−	+	+	+	7.0%	5.0%	Basal-like	Basal-like
1970–1979	3–11	1973	IDC	1	+	+	+	+	+	+	−	−	−	−	−	−	6.0%	4.7%	Luminal A	Luminal A
	3–12	1974	IDC	3	+	+	+	+	+	+	−	−	−	−	−	−	6.0%	7.3%	Luminal A	Luminal A
	3–13	1977	IDC	3	a	+	a	+	a	+	a	−	a	−	a	−	a	16.3%	a	Luminal B
	3–14	1979	Solid papillary carcinoma	2	+	+	+	+	+	+	−	−	+	+	−	−	1.7%	2.5%	Luminal A	Luminal A
	3–15	1974	IDC	3	+	+	−	−	−	−	3+	3+	+	−	−	−	8.7%	8.0%	HER2	HER2
1980–1989	4–16	1988	IDC	3	+	+	+	+	+	+	−	−	−	−	−	−	2.0%	2.0%	Luminal A	Luminal A
	4–17	1980	IDC	3	+	+	−	−	−	−	−	−	−	−	−	−	9.7%	7.3%	Unclassifiable	Unclassifiable
	4–18	1985	IDC	3	+	+	+	+	+	−	−	−	−	−	−	−	10.0%	3.5%	Luminal A	Luminal A
	4–19	1986	Tubular carcinoma	1	+	+	+	+	−	−	−	b	−	−	−	−	1.3%	1.0%	Luminal A	**Unknown**
	4–20	1985	IDC	1	+	+	+	+	−	+	−	−	−	−	−	−	1.0%	0.5%	Luminal A	Luminal A
1990–1999	5–21	1999	IDC	3	+	+	−	−	−	−	3+	3+	−	−	−	−	12.7%	13.3%	HER 2	HER 2
	5–22	1994	Mixed IDC and mucinous carcinoma	3	+	+	+	+	+	+	−	−	−	−	−	−	9.3%	11.0%	Luminal A	Luminal A
	5–23	1996	Invasive carcinoma with ductal and lobular features	2	+	+	+	+	+	+	−	−	−	−	−	−	1.7%	3.5%	Luminal A	Luminal A
	5–24	1995	Invasive carcinoma with ductal and lobular features	2	+	+	+	+	+	+	−	−	−	−	−	−	16.0%	8.0%	Luminal B	Luminal A
	5–25	1998	Invasive carcinoma with ductal and lobular features	2	+	+	+	+	−	−	−	−	−	−	−	−	5.7%	7.7%	Luminal A	Luminal A
2000–2009	6–26	2006	IDC	3	+	+	+	+	+	+	3+	3+	−	−	−	−	17.7%	12.0%	Luminal B	Luminal B
	6–27	2002	IDC	3	+	+	−	−	−	−	3+	3+	−	−	−	−	13.3%	14.0%	HER 2	HER 2
	6–28	2000	Invasive carcinoma with ductal and lobular features	2	+	+	+	+	+	+	−	b	−	−	−	−	5.7%	8.3%	Luminal A	Luminal A or Luminal B
	6–29	2002	IDC with medullary features	3	+	+	−	−	−	−	2+	−	+	+	+	+	6.3%	7.7%	Basal-like	Basal-like
	6–30	2000	IDC	3	+	+	+	+	+	+	−	−	−	−	−	−	6.3%	6.7%	Luminal A	Luminal A
Percent concordance					100%		100%		93%		97%		93%		97%		90%		90%	
Kappa (95% confidence interval)					1.000 (1.000, 1.000)		1.000 (1.000, 1.000)		0.926 (0.705, 1.000)		0.908 (0.731, 1.000)		0.628 (0.155, 1.000)		0.782 (0.372, 1.000)		0.346^c^ (−0.225, 0.916)		0.724 (0.591, 0.976)	

Abbreviations: CK, cytokeratin; ER, estrogen receptor; EGFR, epidermal growth factor receptor; FFPE, formalin-fixed paraffin-embedded; HER2, human epidermal growth factor receptor 2; ID, case identification; IDC, invasive ductal carcinoma; PR, progesterone receptor; TMA, tissue microarray.

Molecular phenotype algorithm^13,19–21^:

Luminal A—ER^+^ and/or PR^+^, HER2^−^, Ki67<14%.

Luminal B—ER^+^ and/or PR^+^ and HER2^+^ OR ER^+^ and/or PR^+^, HER2^−^, and Ki67⩾14%.

HER2—ER^−^ and PR^−^, and HER2^+^.

Basal-like—ER^−^, PR^−^, HER2^−^, CK 5/6^+^ and/or EGFR^+^.

a Results for case 3–13 were missing for TMA1.

b Results unknown owing to no tissue being present in all 3 cores obtained for the TMA.

c Kappa for Ki67 for dichotomous categorization as <14% vs. ⩾14%.
